# *catena*-Poly[[(2,2′:6′,2′′-terpyridine-κ^3^ *N*,*N*′,*N*′′)(tricyano­methanido-κ*N*)nickel(II)]-μ-tricyano­methanido]

**DOI:** 10.1107/S1600536808030377

**Published:** 2008-09-27

**Authors:** Jun Luo, Xin-Rong Zhang, Wei-Quan Dai, Li-Li Cui, Bao-Shu Liu

**Affiliations:** a School of Pharmacy, Second Military Medical University, Shanghai 200433, People’s Republic of China

## Abstract

In the title complex, [Ni(C_4_N_3_)_2_(C_15_H_11_N_3_)]_*n*_, each of the two different Ni^II^ atoms is coordinated by one 2,2′:6′2′′-terpyridine (terpy) and three tricyano­methanide ligands in a distorted octa­hedral geometry. The Ni^II^ atoms are linked to each other, forming an infinite chain parallel to (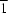
10). π–π Stacking inter­actions of terpy mol­ecules between adjacent chains (centroid–centroid distance = 3.785 Å), along with weak inter­molecular C—H⋯N hydrogen bonds involving the uncoordinated terminal N atoms of the tricyanomethanide ions and the terpyridine H atoms, result in the formation of a three-dimensional network structure.

## Related literature

For general background, see: Abrahams *et al.* (2003 ▶); Batten & Murray (2003 ▶); Batten *et al.* (1998 ▶, 2000 ▶); Feyerherm *et al.* (2003 ▶, 2004 ▶); Manson & Schlueter (2004 ▶); Manson *et al.* (1998 ▶, 2000 ▶); Miller & Manson (2001 ▶); Yuste *et al.* (2008 ▶). For related structures, see: Baker *et al.* (1995 ▶); Batten *et al.* (1999 ▶); Hoshino *et al.* (1999 ▶); Indumathy *et al.* (2007 ▶); Luo *et al.* (2005 ▶); Potočňák *et al.* (2007 ▶).
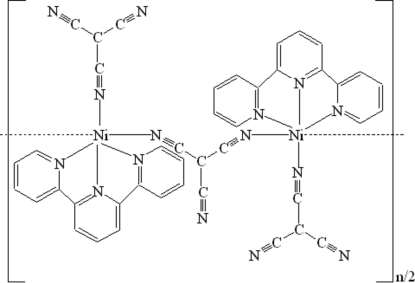


## Experimental

### 

#### Crystal data

[Ni(C_4_N_3_)_2_(C_15_H_11_N_3_)]
*M* *_r_* = 944.24
Triclinic, 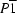

*a* = 8.410 (3) Å
*b* = 15.581 (5) Å
*c* = 16.816 (5) Å
α = 93.762 (4)°
β = 90.110 (4)°
γ = 97.572 (5)°
*V* = 2179.4 (11) Å^3^
*Z* = 2
Mo *K*α radiation
μ = 0.92 mm^−1^
*T* = 293 (2) K
0.20 × 0.15 × 0.15 mm

#### Data collection

Bruker SMART CCD area-detector diffractometer
Absorption correction: multi-scan (*SADABS*; Sheldrick, 1996 ▶) *T*_min_ = 0.837, *T*_max_ = 0.874
10952 measured reflections
9204 independent reflections
6799 reflections with *I* > 2σ(*I*)
*R*_int_ = 0.031

#### Refinement

*R*[*F* ^2^ > 2σ(*F* ^2^)] = 0.053
*wR*(*F* ^2^) = 0.136
*S* = 1.03
9204 reflections
595 parameters
H-atom parameters constrained
Δρ_max_ = 0.51 e Å^−3^
Δρ_min_ = −0.32 e Å^−3^

### 

Data collection: *SMART* (Bruker, 2000 ▶); cell refinement: *SAINT* (Bruker, 2000 ▶); data reduction: *SAINT* (Bruker, 2000 ▶); program(s) used to solve structure: *SHELXS97* (Sheldrick, 2008 ▶); program(s) used to refine structure: *SHELXL97* (Sheldrick, 2008 ▶); molecular graphics: *SHELXTL* (Sheldrick, 2008 ▶); software used to prepare material for publication: *SHELXTL* (Sheldrick, 2008 ▶).

## Supplementary Material

Crystal structure: contains datablocks global, I. DOI: 10.1107/S1600536808030377/dn2372sup1.cif

Structure factors: contains datablocks I. DOI: 10.1107/S1600536808030377/dn2372Isup2.hkl

Additional supplementary materials:  crystallographic information; 3D view; checkCIF report

## Figures and Tables

**Table 1:** Hydrogen-bond geometry (Å, °)

*D*—H⋯*A*	*D*—H	H⋯*A*	*D*⋯*A*	*D*—H⋯*A*
C1—H1⋯N8^i^	0.93	2.61	3.213 (6)	123
C4—H4⋯N6^ii^	0.93	2.59	3.459 (7)	156
C7—H7⋯N6^ii^	0.93	2.55	3.434 (6)	158
C9—H9⋯N17^iii^	0.93	2.60	3.517 (6)	167
C30—H30⋯N18^iv^	0.93	2.60	3.386 (6)	143
C34—H34⋯N11^v^	0.93	2.57	3.467 (6)	162
C40—H40⋯N8^vi^	0.93	2.61	3.292 (7)	130

Symmetry codes: (i) 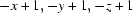; (ii) 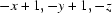; (iii) 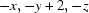; (iv) 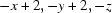; (v) 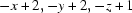; (vi) 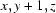.

**Fig. 1.**
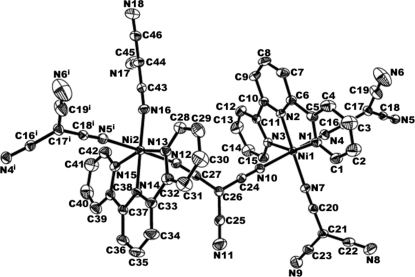
A view of the one-dimensional chain in (I), showing the atom-labelling scheme. Displacement ellipsoids are drawn at the 50% probability level and H atoms have been omitted for clarity [symmetry code: (i) x + 1, y + 1, z].

**Fig. 2.**
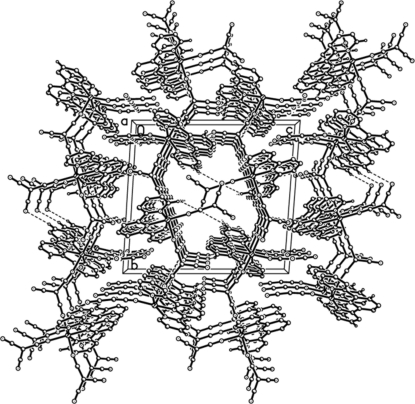
The three-dimensional network structure of (I) formed via terpy π-π interactions and hydrogen bonding interactions, viewed along the a axis.

## supplementary crystallographic information

### Comment

Recently, coordination polymers assembled by tricyanomethanide (tcm) have attracted considerable interest bacause of their novel structure characteristics and fascinating magnetic properties (Batten *et al.*, 2003; Miller *et al.*, 2001; Feyerherm *et al.*, 2003). To our knowledge, most binary tcm complexes display a rutile-like structure (Manson *et al.*, 2000, 1998; Hoshino *et al.*, 1999; Feyerherm *et al.*, 2004), except that a doubly interpenetrated (6,3) sheet was observed in Ag(tcm)_2_ (Abrahams *et al.*, 2003). To clarify the structure-properties relationship of tcm complexes, diverse co-ligands such as hexamethylenetetramine, 4,4-bipyridyl, 1,2-bi(4-pyridyl)ethane were introduced and the structures as well as magnetic properties of the adjusted complexes have been systematically investigated. Among the Cu(I) or Cd(II) tcm complexes with these co-ligands, numerous structure types range from doubly interpenetrated (4,4) sheet to three-dimensional rutile networks were observed (Batten *et al.*, 2000, 1998). By contrast, modification of the Mn(II)-tcm binary system with 4,4-bipyridyl as co-ligands leads to the formation of a one dimensional chain-like structure (Manson *et al.*, 2004). On the other hand, 2,2':6'2''-terpyridine (terpy) is a novel co-ligand and has three potential nitrogen donor atoms. However, only few tcm complexes with terpy as a co-ligand have ever been reported (Yuste *et al.*, 2008). In order to further study the effect of the nature of co-ligands on the structures and properties of tricyanomethanide complexes, we herein report the synthesis and crystal structure of the new tricyanomethanide complex [Ni(terpy)(C_4_N_3_)_2_]_2_ (I).

In (I) the asymmetric unit is built up from two nickel ions: Ni1 and Ni2. Both Ni atoms display a slightly distorted octahedral coordination with the three N atoms of the terpyridine molecule and one terminal tcm N atom forming the equatorial plane, whereas two bridging tcm N atoms are located in axial position .

The distances and angles within the two octahedrons are roughly similar within experimental error. The only significant difference appear in the bending at the N atom of terminal tcm ligand located in the equatorial plane. Indeed, the Ni2-N16-C44 angle, 167.5 (3)°, whereas the corresponding Ni1-N7-C20 angle is 177.6 (5)°. The Ni1 and Ni2 atoms are linked trough a tcm bridge and each dinuclear units are further linked trough symetry related tcm bridges to form an infinite chain structure parallel to the (-1 1 0) plane (Fig. 1).

Intermolecular C-H···N hydrogen bonding between the uncoordinated terminal N atoms of the tcm ions and the H atoms of terpyridine groups (Table 1) and π-π stacking interactions involving terpyridine rings between adjacent chains along with (Table 2), result in the formation of a three-dimensional network structure (Fig. 2).

The Ni—N(terpy) distances (1.985 (3) to 2.110 (3) Å) are almost equal to Ni—N(tcm) distances (2.014 (3) to 2.101 (3) Å), and are respectively comparable to the corresponding distances found in nickel-terpyridine (Baker *et al.*, 1995) and nickel-tcm complexes (Luo *et al.*, 2005; Potocnák *et al.*, 2007).

The bond distances and angles within the terpyridine rings are in the normal ranges observed for terpyridine-containing complexes (Indumathy *et al.*, 2007). Each tricyanomethanide moiety is almost planar and the bond distances and angles are in good agreement with those found in other tricyanomethanide complexes (Hoshino *et al.*, 1999; Batten *et al.*, 1999).

### Experimental

A 5 ml ethanol solution of terpyridine (0.10 mmol, 23.33 mg) and a 2 ml aqueous pale-green solution of nickel nitrate (0.10 mmol, 29.08 mg) were mixed and stirred for 5 min, the mixed solution became yellow. To the mixture was added a 3 ml ethanol-water solution (EtOH:H_2_O = 2:1, V:V) of potassium tricyanomethanide (0.20 mmol, 25.83 mg). After stirring for another 5 min, the yellow solution was filtered and the filtrate was slowly evaporated in air. After two weeks, pale-purple block crystals of (I) were isolated in 23% yield. Anal: Calculated for C46H22N18Ni2: C 58.52%, H 2.35%, N 26.70%. Found C 58.68%, H 2.43%, N 26.84%.

### Refinement

In (I) the terpyridine H atoms were placed in geometrically idealized positions and constrained to ride on their parent C atoms with C—H distances of 0.93 Å and U_iso_(H)=1.2U_eq_(C).

### Crystal data

**Table:**

[Ni(C_4_N_3_)_2_(C_15_H_11_N_3_)]	*Z* = 2
*M**_r_* = 944.24	*F*(000) = 960
Triclinic, *P*1	*D*_x_ = 1.439 Mg m^−^^3^
Hall symbol: -P 1	Mo *K*α radiation, λ = 0.71073 Å
*a* = 8.410 (3) Å	Cell parameters from 944 reflections
*b* = 15.581 (5) Å	θ = 2.6–24.5°
*c* = 16.816 (5) Å	µ = 0.92 mm^−^^1^
α = 93.762 (4)°	*T* = 293 K
β = 90.110 (4)°	Block, pale-purple
γ = 97.572 (5)°	0.20 × 0.15 × 0.15 mm
*V* = 2179.4 (11) Å^3^	

### Data collection

**Table:**

Bruker SMART CCD area-detector diffractometer	9204 independent reflections
Radiation source: fine-focus sealed tube	6799 reflections with *I* > 2σ(*I*)
graphite	*R*_int_ = 0.031
φ and ω scans	θ_max_ = 27.0°, θ_min_ = 1.2°
Absorption correction: multi-scan (SADABS; Sheldrick, 1996)	*h* = −10→8
*T*_min_ = 0.837, *T*_max_ = 0.874	*k* = −19→19
10952 measured reflections	*l* = −21→21

### Refinement

**Table:**

Refinement on *F*^2^	Primary atom site location: structure-invariant direct methods
Least-squares matrix: full	Secondary atom site location: difference Fourier map
*R*[*F*^2^ > 2σ(*F*^2^)] = 0.053	Hydrogen site location: inferred from neighbouring sites
*wR*(*F*^2^) = 0.136	H-atom parameters constrained
*S* = 1.03	*w* = 1/[σ^2^(*F*_o_^2^) + (0.0597*P*)^2^ + 0.2733*P*] where *P* = (*F*_o_^2^ + 2*F*_c_^2^)/3
9204 reflections	(Δ/σ)_max_ = 0.002
595 parameters	Δρ_max_ = 0.51 e Å^−^^3^
0 restraints	Δρ_min_ = −0.32 e Å^−^^3^

### Special details

**Table:**

Geometry. All esds (except the esd in the dihedral angle between two l.s. planes) are estimated using the full covariance matrix. The cell esds are taken into account individually in the estimation of esds in distances, angles and torsion angles; correlations between esds in cell parameters are only used when they are defined by crystal symmetry. An approximate (isotropic) treatment of cell esds is used for estimating esds involving l.s. planes.
Refinement. Refinement of *F*^2^ against ALL reflections. The weighted *R*-factor *wR* and goodness of fit *S* are based on *F*^2^, conventional *R*-factors *R* are based on *F*, with *F* set to zero for negative *F*^2^. The threshold expression of *F*^2^ > σ(*F*^2^) is used only for calculating *R*-factors(gt) *etc*. and is not relevant to the choice of reflections for refinement. *R*-factors based on *F*^2^ are statistically about twice as large as those based on *F*, and *R*- factors based on ALL data will be even larger.

### Fractional atomic coordinates and isotropic or equivalent isotropic displacement parameters (Å^2^)

**Table:**

	*x*	*y*	*z*	*U*_iso_*/*U*_eq_	
Ni1	0.22374 (5)	0.63410 (2)	0.27013 (2)	0.02854 (12)	
Ni2	0.70217 (5)	1.13422 (3)	0.23620 (2)	0.03109 (12)	
N1	0.4308 (3)	0.58137 (18)	0.23108 (17)	0.0379 (6)	
N2	0.2387 (3)	0.66731 (17)	0.15730 (15)	0.0319 (6)	
N3	0.0177 (3)	0.69351 (17)	0.25830 (16)	0.0341 (6)	
N4	0.0978 (4)	0.51428 (19)	0.22881 (18)	0.0425 (7)	
N5	−0.1568 (4)	0.24281 (18)	0.19521 (17)	0.0426 (7)	
N6	0.2572 (6)	0.3427 (3)	0.0496 (3)	0.125 (2)	
N7	0.2082 (4)	0.59875 (18)	0.38324 (17)	0.0418 (7)	
N8	0.3241 (7)	0.4149 (3)	0.5555 (2)	0.0949 (15)	
N9	0.0357 (5)	0.6229 (2)	0.6311 (2)	0.0722 (11)	
N10	0.3504 (3)	0.75359 (18)	0.31275 (16)	0.0384 (7)	
N11	0.6264 (5)	0.9131 (3)	0.5073 (2)	0.0762 (12)	
N12	0.5724 (4)	1.02474 (18)	0.28128 (17)	0.0432 (7)	
N13	0.9001 (3)	1.06682 (18)	0.22472 (16)	0.0369 (6)	
N14	0.7987 (3)	1.15562 (17)	0.34463 (16)	0.0347 (6)	
N15	0.5437 (4)	1.21064 (18)	0.29291 (17)	0.0414 (7)	
N16	0.5894 (4)	1.11062 (19)	0.12874 (17)	0.0424 (7)	
N17	0.1207 (5)	1.1214 (3)	0.0126 (2)	0.0764 (12)	
N18	0.5484 (5)	1.1226 (3)	−0.1334 (2)	0.0696 (11)	
C1	0.5173 (5)	0.5330 (3)	0.2712 (2)	0.0541 (10)	
H1	0.4905	0.5231	0.3238	0.065*	
C2	0.6450 (6)	0.4966 (4)	0.2383 (3)	0.0775 (15)	
H2	0.7041	0.4636	0.2682	0.093*	
C3	0.6826 (6)	0.5102 (4)	0.1607 (3)	0.0785 (15)	
H3	0.7675	0.4861	0.1367	0.094*	
C4	0.5941 (5)	0.5597 (3)	0.1187 (3)	0.0621 (12)	
H4	0.6191	0.5700	0.0660	0.075*	
C5	0.4680 (4)	0.5940 (2)	0.1547 (2)	0.0407 (8)	
C6	0.3607 (4)	0.6445 (2)	0.11328 (19)	0.0367 (8)	
C7	0.3729 (5)	0.6668 (3)	0.0341 (2)	0.0502 (10)	
H7	0.4571	0.6521	0.0024	0.060*	
C8	0.2584 (5)	0.7108 (3)	0.0042 (2)	0.0543 (10)	
H8	0.2651	0.7259	−0.0484	0.065*	
C9	0.1326 (4)	0.7333 (2)	0.0506 (2)	0.0459 (9)	
H9	0.0552	0.7637	0.0303	0.055*	
C10	0.1261 (4)	0.7091 (2)	0.1281 (2)	0.0375 (8)	
C11	−0.0013 (4)	0.7247 (2)	0.1860 (2)	0.0351 (7)	
C12	−0.1317 (5)	0.7642 (3)	0.1692 (2)	0.0526 (10)	
H12	−0.1433	0.7854	0.1194	0.063*	
C13	−0.2460 (5)	0.7723 (3)	0.2268 (3)	0.0673 (13)	
H13	−0.3348	0.7997	0.2163	0.081*	
C14	−0.2284 (5)	0.7398 (3)	0.2996 (3)	0.0619 (12)	
H14	−0.3050	0.7441	0.3389	0.074*	
C15	−0.0952 (5)	0.7010 (2)	0.3127 (2)	0.0472 (9)	
H15	−0.0828	0.6788	0.3620	0.057*	
C16	0.0740 (4)	0.4459 (2)	0.1991 (2)	0.0350 (7)	
C17	0.0544 (4)	0.3643 (2)	0.1586 (2)	0.0409 (8)	
C18	−0.0617 (4)	0.2975 (2)	0.17997 (19)	0.0353 (7)	
C19	0.1645 (6)	0.3509 (3)	0.0985 (3)	0.0687 (14)	
C20	0.2002 (4)	0.5758 (2)	0.4466 (2)	0.0382 (8)	
C21	0.1934 (5)	0.5477 (2)	0.5238 (2)	0.0428 (9)	
C22	0.2664 (6)	0.4744 (3)	0.5412 (2)	0.0597 (11)	
C23	0.1061 (5)	0.5892 (2)	0.5829 (2)	0.0485 (9)	
C24	0.4178 (4)	0.8188 (2)	0.33652 (19)	0.0332 (7)	
C25	0.5722 (5)	0.9050 (2)	0.4446 (2)	0.0463 (9)	
C26	0.5053 (4)	0.8968 (2)	0.3671 (2)	0.0378 (8)	
C27	0.5407 (4)	0.9663 (2)	0.3185 (2)	0.0350 (7)	
C28	0.9457 (5)	1.0230 (2)	0.1600 (2)	0.0485 (9)	
H28	0.8846	1.0212	0.1136	0.058*	
C29	1.0777 (5)	0.9805 (3)	0.1583 (3)	0.0605 (11)	
H29	1.1036	0.9491	0.1123	0.073*	
C30	1.1719 (5)	0.9848 (3)	0.2259 (3)	0.0731 (14)	
H30	1.2636	0.9573	0.2260	0.088*	
C31	1.1276 (5)	1.0309 (3)	0.2938 (3)	0.0671 (13)	
H31	1.1894	1.0351	0.3401	0.081*	
C32	0.9911 (4)	1.0703 (2)	0.2916 (2)	0.0442 (9)	
C33	0.9294 (4)	1.1198 (2)	0.3613 (2)	0.0430 (8)	
C34	0.9940 (5)	1.1274 (3)	0.4385 (2)	0.0647 (12)	
H34	1.0863	1.1035	0.4501	0.078*	
C35	0.9155 (6)	1.1716 (3)	0.4966 (2)	0.0680 (13)	
H35	0.9547	1.1773	0.5487	0.082*	
C36	0.7803 (6)	1.2073 (3)	0.4785 (2)	0.0565 (11)	
H36	0.7277	1.2367	0.5182	0.068*	
C37	0.7217 (4)	1.1993 (2)	0.4000 (2)	0.0386 (8)	
C38	0.5795 (4)	1.2319 (2)	0.3709 (2)	0.0406 (8)	
C39	0.4833 (5)	1.2792 (3)	0.4179 (3)	0.0574 (11)	
H39	0.5097	1.2950	0.4710	0.069*	
C40	0.3456 (6)	1.3029 (3)	0.3838 (3)	0.0734 (14)	
H40	0.2778	1.3335	0.4148	0.088*	
C41	0.3101 (6)	1.2817 (3)	0.3061 (3)	0.0743 (14)	
H41	0.2189	1.2976	0.2828	0.089*	
C42	0.4132 (5)	1.2357 (3)	0.2622 (3)	0.0587 (11)	
H42	0.3900	1.2216	0.2084	0.070*	
C43	0.5134 (4)	1.1105 (2)	0.0723 (2)	0.0392 (8)	
C44	0.4195 (5)	1.1110 (2)	0.0033 (2)	0.0454 (9)	
C45	0.2561 (5)	1.1160 (3)	0.0093 (2)	0.0504 (10)	
C46	0.4922 (5)	1.1164 (2)	−0.0719 (2)	0.0499 (10)	

### Atomic displacement parameters (Å^2^)

**Table:**

	*U*^11^	*U*^22^	*U*^33^	*U*^12^	*U*^13^	*U*^23^
Ni1	0.0306 (2)	0.0289 (2)	0.0255 (2)	0.00083 (17)	0.00230 (16)	0.00317 (16)
Ni2	0.0340 (2)	0.0308 (2)	0.0267 (2)	−0.00316 (17)	−0.00293 (17)	0.00346 (16)
N1	0.0352 (15)	0.0441 (17)	0.0344 (15)	0.0066 (13)	−0.0017 (12)	0.0014 (13)
N2	0.0316 (14)	0.0360 (15)	0.0277 (14)	0.0014 (12)	−0.0003 (11)	0.0058 (11)
N3	0.0313 (14)	0.0343 (15)	0.0368 (15)	0.0042 (12)	0.0032 (12)	0.0033 (12)
N4	0.0445 (17)	0.0367 (17)	0.0432 (17)	−0.0051 (13)	0.0066 (13)	0.0017 (13)
N5	0.0514 (18)	0.0373 (16)	0.0354 (16)	−0.0084 (14)	0.0021 (14)	0.0037 (13)
N6	0.121 (4)	0.106 (4)	0.132 (5)	−0.025 (3)	0.088 (4)	−0.033 (3)
N7	0.0541 (19)	0.0410 (16)	0.0310 (16)	0.0059 (14)	0.0054 (13)	0.0086 (13)
N8	0.155 (5)	0.093 (3)	0.049 (2)	0.056 (3)	0.000 (3)	0.016 (2)
N9	0.098 (3)	0.065 (2)	0.052 (2)	0.004 (2)	0.029 (2)	0.0060 (18)
N10	0.0433 (17)	0.0352 (16)	0.0343 (15)	−0.0040 (13)	−0.0001 (13)	0.0020 (12)
N11	0.088 (3)	0.073 (3)	0.064 (3)	−0.008 (2)	−0.028 (2)	0.014 (2)
N12	0.0507 (18)	0.0376 (16)	0.0369 (16)	−0.0125 (14)	−0.0061 (14)	0.0060 (13)
N13	0.0350 (15)	0.0400 (16)	0.0345 (15)	0.0005 (12)	−0.0042 (12)	0.0028 (12)
N14	0.0371 (15)	0.0346 (15)	0.0295 (14)	−0.0047 (12)	−0.0033 (12)	−0.0003 (11)
N15	0.0477 (18)	0.0407 (16)	0.0359 (16)	0.0052 (14)	0.0040 (13)	0.0050 (13)
N16	0.0453 (17)	0.0475 (17)	0.0330 (16)	−0.0010 (14)	−0.0077 (13)	0.0064 (13)
N17	0.064 (3)	0.098 (3)	0.070 (3)	0.009 (2)	−0.010 (2)	0.023 (2)
N18	0.087 (3)	0.080 (3)	0.040 (2)	0.010 (2)	0.0002 (19)	−0.0033 (18)
C1	0.054 (2)	0.067 (3)	0.044 (2)	0.020 (2)	−0.0013 (19)	0.0052 (19)
C2	0.069 (3)	0.110 (4)	0.063 (3)	0.051 (3)	−0.004 (2)	0.008 (3)
C3	0.060 (3)	0.116 (4)	0.068 (3)	0.045 (3)	0.012 (2)	−0.003 (3)
C4	0.045 (2)	0.097 (3)	0.047 (2)	0.023 (2)	0.0106 (19)	0.002 (2)
C5	0.0359 (18)	0.049 (2)	0.0357 (19)	0.0016 (16)	0.0041 (15)	−0.0015 (16)
C6	0.0363 (18)	0.0403 (19)	0.0322 (17)	0.0000 (15)	0.0046 (14)	0.0036 (14)
C7	0.050 (2)	0.065 (3)	0.0334 (19)	−0.002 (2)	0.0145 (17)	0.0046 (18)
C8	0.066 (3)	0.065 (3)	0.0304 (19)	−0.004 (2)	0.0008 (18)	0.0132 (18)
C9	0.044 (2)	0.057 (2)	0.039 (2)	0.0057 (18)	−0.0077 (16)	0.0151 (17)
C10	0.0379 (18)	0.0385 (18)	0.0343 (18)	−0.0035 (15)	−0.0001 (15)	0.0057 (14)
C11	0.0352 (18)	0.0332 (17)	0.0367 (18)	0.0029 (14)	−0.0039 (14)	0.0033 (14)
C12	0.049 (2)	0.058 (2)	0.055 (2)	0.0158 (19)	−0.0030 (19)	0.0154 (19)
C13	0.047 (2)	0.089 (3)	0.075 (3)	0.036 (2)	0.002 (2)	0.017 (3)
C14	0.043 (2)	0.087 (3)	0.062 (3)	0.031 (2)	0.014 (2)	0.010 (2)
C15	0.048 (2)	0.054 (2)	0.041 (2)	0.0088 (18)	0.0094 (17)	0.0064 (17)
C16	0.0310 (17)	0.0373 (19)	0.0356 (18)	−0.0018 (14)	0.0028 (14)	0.0081 (15)
C17	0.045 (2)	0.0344 (18)	0.0397 (19)	−0.0055 (15)	0.0071 (16)	−0.0012 (15)
C18	0.0408 (19)	0.0327 (17)	0.0304 (17)	−0.0012 (15)	−0.0020 (14)	−0.0004 (14)
C19	0.073 (3)	0.053 (3)	0.072 (3)	−0.014 (2)	0.031 (3)	−0.012 (2)
C20	0.0369 (18)	0.0367 (18)	0.039 (2)	−0.0009 (15)	−0.0004 (15)	0.0003 (15)
C21	0.055 (2)	0.044 (2)	0.0279 (17)	−0.0007 (17)	0.0034 (16)	0.0066 (15)
C22	0.086 (3)	0.066 (3)	0.028 (2)	0.010 (3)	0.003 (2)	0.0058 (19)
C23	0.058 (2)	0.047 (2)	0.037 (2)	−0.0065 (19)	0.0036 (18)	0.0083 (17)
C24	0.0309 (17)	0.0364 (18)	0.0330 (17)	0.0052 (14)	0.0061 (14)	0.0071 (14)
C25	0.047 (2)	0.040 (2)	0.050 (2)	−0.0037 (17)	−0.0137 (18)	0.0090 (17)
C26	0.0402 (19)	0.0316 (17)	0.0391 (19)	−0.0042 (15)	−0.0014 (15)	0.0028 (14)
C27	0.0303 (17)	0.0349 (18)	0.0369 (18)	−0.0029 (14)	−0.0043 (14)	−0.0050 (15)
C28	0.049 (2)	0.052 (2)	0.043 (2)	0.0051 (18)	−0.0011 (17)	−0.0005 (18)
C29	0.054 (3)	0.059 (3)	0.068 (3)	0.011 (2)	0.005 (2)	−0.009 (2)
C30	0.049 (3)	0.089 (4)	0.084 (4)	0.028 (2)	−0.006 (2)	−0.012 (3)
C31	0.045 (2)	0.085 (3)	0.073 (3)	0.020 (2)	−0.018 (2)	−0.007 (3)
C32	0.0366 (19)	0.049 (2)	0.045 (2)	0.0007 (17)	−0.0050 (16)	0.0018 (17)
C33	0.042 (2)	0.046 (2)	0.039 (2)	0.0000 (17)	−0.0084 (16)	0.0027 (16)
C34	0.063 (3)	0.084 (3)	0.048 (2)	0.013 (2)	−0.026 (2)	0.000 (2)
C35	0.080 (3)	0.083 (3)	0.037 (2)	0.004 (3)	−0.022 (2)	−0.008 (2)
C36	0.078 (3)	0.053 (2)	0.033 (2)	−0.007 (2)	0.003 (2)	−0.0044 (17)
C37	0.046 (2)	0.0333 (18)	0.0327 (18)	−0.0082 (15)	0.0027 (15)	0.0003 (14)
C38	0.047 (2)	0.0339 (18)	0.0391 (19)	−0.0024 (16)	0.0062 (16)	0.0027 (15)
C39	0.066 (3)	0.052 (2)	0.052 (2)	0.007 (2)	0.011 (2)	−0.0078 (19)
C40	0.072 (3)	0.070 (3)	0.082 (4)	0.026 (3)	0.019 (3)	−0.006 (3)
C41	0.071 (3)	0.086 (4)	0.073 (3)	0.037 (3)	−0.006 (3)	−0.003 (3)
C42	0.063 (3)	0.064 (3)	0.053 (2)	0.023 (2)	−0.005 (2)	0.002 (2)
C43	0.049 (2)	0.0325 (18)	0.0352 (19)	0.0032 (16)	−0.0038 (16)	0.0017 (15)
C44	0.055 (2)	0.047 (2)	0.0343 (19)	0.0079 (18)	−0.0129 (17)	0.0031 (16)
C45	0.058 (3)	0.054 (2)	0.039 (2)	0.006 (2)	−0.0147 (19)	0.0098 (17)
C46	0.062 (3)	0.049 (2)	0.039 (2)	0.0087 (19)	−0.0144 (19)	−0.0011 (17)

### Geometric parameters (Å, °)

**Table:**

Ni1—N2	1.999 (3)	C8—H8	0.9300
Ni1—N7	2.014 (3)	C9—C10	1.380 (5)
Ni1—N3	2.085 (3)	C9—H9	0.9300
Ni1—N4	2.097 (3)	C10—C11	1.482 (5)
Ni1—N10	2.101 (3)	C11—C12	1.364 (5)
Ni1—N1	2.110 (3)	C12—C13	1.378 (6)
Ni2—N14	1.984 (3)	C12—H12	0.9300
Ni2—N16	2.028 (3)	C13—C14	1.369 (6)
Ni2—N13	2.085 (3)	C13—H13	0.9300
Ni2—N12	2.088 (3)	C14—C15	1.365 (5)
Ni2—N5^i^	2.091 (3)	C14—H14	0.9300
Ni2—N15	2.092 (3)	C15—H15	0.9300
N1—C1	1.327 (5)	C16—C17	1.392 (5)
N1—C5	1.344 (4)	C17—C18	1.395 (5)
N2—C10	1.328 (4)	C17—C19	1.397 (5)
N2—C6	1.338 (4)	C20—C21	1.395 (5)
N3—C15	1.331 (4)	C21—C23	1.409 (5)
N3—C11	1.355 (4)	C21—C22	1.413 (6)
N4—C16	1.140 (4)	C24—C26	1.401 (4)
N5—C18	1.132 (4)	C25—C26	1.411 (5)
N5—Ni2^ii^	2.091 (3)	C26—C27	1.400 (5)
N6—C19	1.146 (5)	C28—C29	1.365 (5)
N7—C20	1.146 (4)	C28—H28	0.9300
N8—C22	1.140 (6)	C29—C30	1.377 (6)
N9—C23	1.145 (5)	C29—H29	0.9300
N10—C24	1.143 (4)	C30—C31	1.387 (6)
N11—C25	1.139 (5)	C30—H30	0.9300
N12—C27	1.141 (4)	C31—C32	1.373 (5)
N13—C28	1.332 (4)	C31—H31	0.9300
N13—C32	1.353 (4)	C32—C33	1.491 (5)
N14—C33	1.335 (5)	C33—C34	1.397 (5)
N14—C37	1.335 (4)	C34—C35	1.378 (6)
N15—C42	1.328 (5)	C34—H34	0.9300
N15—C38	1.355 (4)	C35—C36	1.372 (6)
N16—C43	1.143 (4)	C35—H35	0.9300
N17—C45	1.154 (5)	C36—C37	1.400 (5)
N18—C46	1.142 (5)	C36—H36	0.9300
C1—C2	1.379 (6)	C37—C38	1.456 (5)
C1—H1	0.9300	C38—C39	1.380 (5)
C2—C3	1.368 (6)	C39—C40	1.393 (6)
C2—H2	0.9300	C39—H39	0.9300
C3—C4	1.366 (6)	C40—C41	1.350 (7)
C3—H3	0.9300	C40—H40	0.9300
C4—C5	1.374 (5)	C41—C42	1.381 (6)
C4—H4	0.9300	C41—H41	0.9300
C5—C6	1.474 (5)	C42—H42	0.9300
C6—C7	1.400 (5)	C43—C44	1.403 (5)
C7—C8	1.366 (6)	C44—C45	1.391 (6)
C7—H7	0.9300	C44—C46	1.408 (5)
C8—C9	1.385 (5)		
			
N2—Ni1—N7	179.14 (11)	N3—C11—C12	121.2 (3)
N2—Ni1—N3	78.14 (11)	N3—C11—C10	114.6 (3)
N7—Ni1—N3	102.22 (12)	C12—C11—C10	124.3 (3)
N2—Ni1—N4	88.50 (11)	C11—C12—C13	119.2 (4)
N7—Ni1—N4	90.72 (12)	C11—C12—H12	120.4
N3—Ni1—N4	90.48 (12)	C13—C12—H12	120.4
N2—Ni1—N10	92.11 (11)	C14—C13—C12	119.8 (4)
N7—Ni1—N10	88.67 (11)	C14—C13—H13	120.1
N3—Ni1—N10	89.83 (11)	C12—C13—H13	120.1
N4—Ni1—N10	179.36 (12)	C15—C14—C13	118.1 (4)
N2—Ni1—N1	78.21 (11)	C15—C14—H14	120.9
N7—Ni1—N1	101.39 (12)	C13—C14—H14	120.9
N3—Ni1—N1	156.10 (11)	N3—C15—C14	123.1 (4)
N4—Ni1—N1	85.56 (11)	N3—C15—H15	118.5
N10—Ni1—N1	94.38 (11)	C14—C15—H15	118.5
N14—Ni2—N16	176.27 (12)	N4—C16—C17	175.5 (4)
N14—Ni2—N13	78.68 (11)	C16—C17—C18	122.2 (3)
N16—Ni2—N13	103.93 (12)	C16—C17—C19	116.2 (3)
N14—Ni2—N12	85.18 (11)	C18—C17—C19	121.5 (3)
N16—Ni2—N12	92.16 (11)	N5—C18—C17	178.2 (4)
N13—Ni2—N12	88.96 (12)	N6—C19—C17	177.7 (5)
N14—Ni2—N5^i^	92.12 (11)	N7—C20—C21	179.0 (4)
N16—Ni2—N5^i^	90.61 (11)	C20—C21—C23	120.1 (4)
N13—Ni2—N5^i^	88.68 (12)	C20—C21—C22	119.6 (3)
N12—Ni2—N5^i^	176.72 (12)	C23—C21—C22	120.2 (3)
N14—Ni2—N15	78.53 (12)	N8—C22—C21	179.4 (6)
N16—Ni2—N15	98.84 (12)	N9—C23—C21	179.7 (5)
N13—Ni2—N15	157.21 (11)	N10—C24—C26	177.6 (4)
N12—Ni2—N15	89.09 (12)	N11—C25—C26	178.8 (4)
N5^i^—Ni2—N15	92.20 (12)	C27—C26—C24	120.4 (3)
C1—N1—C5	118.4 (3)	C27—C26—C25	118.1 (3)
C1—N1—Ni1	127.3 (3)	C24—C26—C25	121.2 (3)
C5—N1—Ni1	114.1 (2)	N12—C27—C26	177.5 (3)
C10—N2—C6	122.4 (3)	N13—C28—C29	123.2 (4)
C10—N2—Ni1	119.2 (2)	N13—C28—H28	118.4
C6—N2—Ni1	118.3 (2)	C29—C28—H28	118.4
C15—N3—C11	118.6 (3)	C28—C29—C30	119.0 (4)
C15—N3—Ni1	126.8 (3)	C28—C29—H29	120.5
C11—N3—Ni1	114.6 (2)	C30—C29—H29	120.5
C16—N4—Ni1	159.6 (3)	C29—C30—C31	118.8 (4)
C18—N5—Ni2^ii^	169.1 (3)	C29—C30—H30	120.6
C20—N7—Ni1	177.6 (3)	C31—C30—H30	120.6
C24—N10—Ni1	179.2 (3)	C32—C31—C30	119.0 (4)
C27—N12—Ni2	160.4 (3)	C32—C31—H31	120.5
C28—N13—C32	118.1 (3)	C30—C31—H31	120.5
C28—N13—Ni2	127.5 (2)	N13—C32—C31	121.9 (4)
C32—N13—Ni2	114.5 (2)	N13—C32—C33	114.2 (3)
C33—N14—C37	122.5 (3)	C31—C32—C33	123.9 (4)
C33—N14—Ni2	119.0 (2)	N14—C33—C34	121.0 (4)
C37—N14—Ni2	118.3 (2)	N14—C33—C32	113.6 (3)
C42—N15—C38	118.8 (3)	C34—C33—C32	125.4 (4)
C42—N15—Ni2	127.5 (3)	C35—C34—C33	117.4 (4)
C38—N15—Ni2	113.6 (2)	C35—C34—H34	121.3
C43—N16—Ni2	167.5 (3)	C33—C34—H34	121.3
N1—C1—C2	122.9 (4)	C36—C35—C34	120.8 (4)
N1—C1—H1	118.6	C36—C35—H35	119.6
C2—C1—H1	118.6	C34—C35—H35	119.6
C3—C2—C1	118.4 (4)	C35—C36—C37	119.7 (4)
C3—C2—H2	120.8	C35—C36—H36	120.2
C1—C2—H2	120.8	C37—C36—H36	120.2
C4—C3—C2	119.2 (4)	N14—C37—C36	118.7 (4)
C4—C3—H3	120.4	N14—C37—C38	114.5 (3)
C2—C3—H3	120.4	C36—C37—C38	126.8 (3)
C3—C4—C5	119.6 (4)	N15—C38—C39	121.0 (4)
C3—C4—H4	120.2	N15—C38—C37	114.9 (3)
C5—C4—H4	120.2	C39—C38—C37	124.1 (4)
N1—C5—C4	121.5 (4)	C38—C39—C40	118.5 (4)
N1—C5—C6	114.9 (3)	C38—C39—H39	120.7
C4—C5—C6	123.6 (3)	C40—C39—H39	120.7
N2—C6—C7	119.2 (3)	C41—C40—C39	120.4 (4)
N2—C6—C5	114.4 (3)	C41—C40—H40	119.8
C7—C6—C5	126.3 (3)	C39—C40—H40	119.8
C8—C7—C6	118.5 (4)	C40—C41—C42	118.1 (5)
C8—C7—H7	120.7	C40—C41—H41	121.0
C6—C7—H7	120.7	C42—C41—H41	121.0
C7—C8—C9	121.3 (3)	N15—C42—C41	123.2 (4)
C7—C8—H8	119.3	N15—C42—H42	118.4
C9—C8—H8	119.3	C41—C42—H42	118.4
C10—C9—C8	117.6 (4)	N16—C43—C44	179.5 (4)
C10—C9—H9	121.2	C45—C44—C43	120.2 (3)
C8—C9—H9	121.2	C45—C44—C46	119.0 (3)
N2—C10—C9	120.9 (3)	C43—C44—C46	120.4 (4)
N2—C10—C11	113.5 (3)	N17—C45—C44	178.3 (5)
C9—C10—C11	125.6 (3)	N18—C46—C44	178.0 (5)

Symmetry codes: (i) *x*+1, *y*+1, *z*; (ii) *x*−1, *y*−1, *z*.

### Hydrogen-bond geometry (Å, °)

**Table:**

*D*—H···*A*	*D*—H	H···*A*	*D*···*A*	*D*—H···*A*
C1—H1···N8^iii^	0.93	2.61	3.213 (6)	123.
C4—H4···N6^iv^	0.93	2.59	3.459 (7)	156.
C7—H7···N6^iv^	0.93	2.55	3.434 (6)	158.
C9—H9···N17^v^	0.93	2.60	3.517 (6)	167.
C30—H30···N18^vi^	0.93	2.60	3.386 (6)	143.
C34—H34···N11^vii^	0.93	2.57	3.467 (6)	162.
C40—H40···N8^viii^	0.93	2.61	3.292 (7)	130.

Symmetry codes: (iii) −*x*+1, −*y*+1, −*z*+1; (iv) −*x*+1, −*y*+1, −*z*; (v) −*x*, −*y*+2, −*z*; (vi) −*x*+2, −*y*+2, −*z*; (vii) −*x*+2, −*y*+2, −*z*+1; (viii) *x*, *y*+1, *z*.

### Table 2 π–π Interaction between some of the terpyridine rings

(Symmetry code: (i) 1+x, y, z)

**Table:**

				
				
Ring 1	Ring 2^i^	Cg1 to Cg2 (Å)	Plane to plane (Å)	Offset (°)
N1 C1···C5	N3 C11···C15	3.785	3.67	13.9
